# Extracellular Vesicles from Skeletal Muscle Cells Efficiently Promote Myogenesis in Induced Pluripotent Stem Cells

**DOI:** 10.3390/cells9061527

**Published:** 2020-06-23

**Authors:** Denisa Baci, Maila Chirivì, Valentina Pace, Fabio Maiullari, Marika Milan, Andrea Rampin, Paolo Somma, Dario Presutti, Silvia Garavelli, Antonino Bruno, Stefano Cannata, Chiara Lanzuolo, Cesare Gargioli, Roberto Rizzi, Claudia Bearzi

**Affiliations:** 1 Institute of Biochemistry and Cell Biology, National Research Council, 00015 Rome, Italy; denisa.baci@uninsubria.it (D.B.); maila.chirivi@yahoo.it (M.C.); va.pace3@gmail.com (V.P.); marika.milan@ibbc.cnr.it (M.M.); rampin88@gmail.com (A.R.); presuttidario@gmail.com (D.P.); 2 Department of Biotechnology and Life Sciences, University of Insubria, 21100 Varese, Italy; 3 Gemelli Molise Hospital, 86100 Campobasso, Italy; fabio.maiullari3d@gmail.com; 4 Flow Cytometry Core, Humanitas Clinical and Research Center, 20089 Milan, Italy; paolo.somma@humanitasresearch.it; 5 Institute for Endocrinology and Oncology “Gaetano Salvatore”, National Research Council, 80131 Naples, Italy; silvia.garavelli@gmail.com; 6 IRCCS MultiMedica, 20138 Milan, Italy; antonino.bruno@multimedica.it; 7 Department of Biology, University of Rome Tor Vergata, 00133 Rome, Italy; cannata@uniroma2.it (S.C.); cesare.gargioli@uniroma2.it (C.G.); 8 Institute of Biomedical Technologies, National Research Council, 20090 Milan, Italy; chiara.lanzuolo@cnr.it; 9 Fondazione Istituto Nazionale di Genetica Molecolare, 20122 Milan, Italy

**Keywords:** iPSC, extracellular vesicles, pericytes, skeletal muscle

## Abstract

The recent advances, offered by cell therapy in the regenerative medicine field, offer a revolutionary potential for the development of innovative cures to restore compromised physiological functions or organs. Adult myogenic precursors, such as myoblasts or satellite cells, possess a marked regenerative capacity, but the exploitation of this potential still encounters significant challenges in clinical application, due to low rate of proliferation in vitro, as well as a reduced self-renewal capacity. In this scenario, induced pluripotent stem cells (iPSCs) can offer not only an inexhaustible source of cells for regenerative therapeutic approaches, but also a valuable alternative for in vitro modeling of patient-specific diseases. In this study we established a reliable protocol to induce the myogenic differentiation of iPSCs, generated from pericytes and fibroblasts, exploiting skeletal muscle-derived extracellular vesicles (EVs), in combination with chemically defined factors. This genetic integration-free approach generates functional skeletal myotubes maintaining the engraftment ability in vivo. Our results demonstrate evidence that EVs can act as biological “shuttles” to deliver specific bioactive molecules for a successful transgene-free differentiation offering new opportunities for disease modeling and regenerative approaches.

## 1. Introduction

Skeletal muscle is a dynamic tissue with remarkable features for endogenous regeneration provided by muscle progenitors, such as satellite cells. However, in the presence of progressive muscle loss or degeneration, such as muscular dystrophies or aging, satellite cell function is largely affected due to an incorrect asymmetric division or aberrant transcriptional regulation [1,2,3,4]. Other adult progenitor cells with myogenic properties, including mesangioblasts [5], pericytes [6,7], muscle side cell population [8], interstitial cells PW1^+^/PAX7^−^ [9], or stem cells derived from bone marrow [10] would be considered promising candidates for muscle repair therapy. Despite this, the reduction of proliferative capacity after isolation and the progressive loss of self-renewal potential strongly limit the use of adult progenitor cells for clinical application [11].

On the other hand, induced pluripotent stem cells (iPSCs) represent a valuable source of myogenic progenitors (MPs), essential for cell-based therapy. Indeed, iPSCs not only would allow autologous transplantation but they can also be produced in large quantities, with an unlimited replication ability in vitro. Furthermore, differentiated iPSCs can be used as individual-specific tissue modeling for the validation of innovative therapies, limiting the toxic effects for the patient and providing early indications on the efficacy [12,13].

Many efforts have been made to establish efficient methods for obtaining MPs from iPSCs, mostly relying on the transgenic expression of major myogenesis regulators, such as myoblast determination protein 1 (MyoD) and Pax7 (key myogenic transcription factors) [14,15,16,17]. The main disadvantage of these approaches is that forced expression of the MyoD protein leads to cell cycle arrest along with the consequent loss of the in vitro muscle progenitor generation. The risk of unwanted genetic recombination is a widespread limiting issue for future clinical application.

The use of chemical modulators to activate relevant myogenic pathways represents a promising approach to enhance the efficiency of myogenic iPSC differentiation [18,19,20]. In particular, a myogenic differentiation improvement of human ESC/iPSC through the treatment with a homologous wingless and Int-1 (Wnt) agonist, the glycogen synthase kinase-3 inhibitor (GSK-3, CHIR9902), has been reported [18,19]. Early inhibition of GSK3β is mandatory for the induction of paraxial mesoderm and activation of the myogenic program [21].

In this study we explored the possibility to exploit the content of extracellular vesicles (EVs), released from differentiated myotubes (MTs), in combination with GSK3 inhibitor, in order to synergistically enhance myogenic differentiation.

To date, the scientific interest regarding the role of EVs in cell-to-cell communication, both in physiological and pathological conditions, is rapidly increasing. EVs are similar in composition to their cell of origin, and their cargo can activate signaling pathways in target cells, thus modulating their activities. In particular, the content of EVs derived from skeletal muscle plays a fundamental role for skeletal muscle homeostasis and development [22,23]. Several studies have shown that skeletal muscle cells release protein/nucleic acid complexes within microvesicles, which promote myogenesis and muscle regeneration [24,25,26,27]. EVs derived from MTs (MT-derived EVs) were found to be able to promote the differentiation of myoblasts by altering the expression of cyclin-D1 and myogenin [27]. Another study reported that exosomes, a subclass of EVs measuring approximately 100 nm in diameter, secreted during myotube differentiation, contribute significantly to the myogenic differentiation of stem cells derived from human adipose tissue [28].

Previous researches have shown that iPSCs retain molecular characteristics of the cell from which they originate, named ‘epigenetic memory’, which is able to strengthen the propensity for re-differentiation in the same tissue [29,30]. On the basis of this, in order to enhance muscle differentiation and exploit myogenic predisposition, muscle-derived pericytes (PCs) and skin fibroblasts (FBs) derived from the same donor were employed as cell sources for iPSC generation. PCs surround the endothelial layer of small/medium vessels that reside beneath the microvascular basement membrane. Despite their role in regulating blood flow, angiogenesis, and maintenance of vascular tissue homeostasis [31], not much is known about pericytes as a source of muscle progenitor cells [6,7]. However, several studies have shown that pericytes are strongly predisposed to differentiate into myogenic lineage and repair muscle damage [6,7,32].

In this study, we established a defined transgene-free protocol, which allows iPSCs, derived either from muscular pericytes or skin fibroblasts, to differentiate into MT-like cells when exposed to GSK-3 inhibitor and EV cargo. This combination improved the differentiation yield into muscle cells up to 70% and the fusion index. After 30 days, evidence of an enhanced muscle differentiation was further revealed by an increased expression of myogenic markers. Furthermore, we found a propensity in pericyte-derived iPSCs to re-differentiate toward the skeletal muscular fate compared to fibroblast-derived counterpart.

Finally, in a pilot study, differentiated iPSCs were injected intramuscularly into anterior tibialis (TA) muscle of immunodeficient alpha-sarcoglycan knockout (KO) mice. The differentiated cells were able to integrate into the host regenerating myofibers, revealing a possible application of the proposed method in regenerative medicine.

## 2. Materials and Methods

### 2.1. Cell Isolation

Skin and muscle specimens were obtained from 3 healthy donors, aged between 20 and 40, upon informed consent in line with the Declaration of Helsinki. Tissues were digested and muscular cell suspension was cultured in alpha Minimum Essential Medium (αMEM; Thermo Fisher Scientific, Waltham, MA, USA), 20% fetal bovine serum (FBS; Thermo Fisher Scientific), and penicillin (100 U/mL; Thermo Fisher Scientific) and streptomycin (100 µg/mL; Thermo Fisher Scientific). Pericytes were then selected by their ability to grow on plastic at low confluence (0.1–1 × 10^4^ cell/cm^2^). Skin cells were plated in Dulbecco’s Modified Eagle Medium (DMEM; Thermo Fisher Scientific) supplemented with 10% FBS, 0.5 mM β-mercaptoethanol (Thermo Fisher Scientific), 100 U/mL penicillin, and 100 µg/mL streptomycin (Thermo Fisher Scientific) at a density of 5 × 10^4^ cells/cm^2^.

### 2.2. Tube-Formation Assay

Pericytes and human umbilical vein endothelial cells (HUVEC; Lonza, Basel, Switzerland) were seeded in 8-well Permanox chamber slides coated with Matrigel (Becton Dickinson Franklin Lakes, NJ, USA), either separately (3.75 × 10^4^ cells per well) or co-cultured together at a 1:4 ratio, in Endothelial Cell Growth Basal Medium-2 (EBM-2; Lonza). Cells were incubated for 5 h to allow tube formation. Images of newly formed networks were captured at 10X magnification.

All experiments were performed in duplicates. Analysis was achieved using the Angiogenesis Analyzer tool (ImageJ Software, https://imagej.nih.gov/ij/).

### 2.3. Pericyte and C2C12 Myogenic Differentiation

Spontaneous skeletal myogenic differentiation of human pericytes and C2C12 cells was induced by plating 10^4^cells/cm^2^ in αMEM, 20% FBS and penicillin/streptomycin. After the cells reached confluence, we replaced the medium with low-serum medium (2% horse serum, HS, Thermo Fisher Scientific) for about 10 (pericyte differentiation) and 5 (C2C12 differentiation) days.

### 2.4. Lentiviral Vector Generation

The lentiviral vector employed for the induction of reprogramming was composed of a single excisable polycistronic lentiviral stem cell cassette (STEMCCA), encoding the Yamanaka factors [33]. Low passage 293T cells (Cell Biolabs, San Diego, CA, USA) were used to produce lentiviruses, employing the psPAX2 and vesicular stomatitis virus G protein (VSV-G) packaging constructs and a calcium phosphate transfection protocol. Supernatants containing STEMCCA lentiviruses were collected 48 h later, filtered, and used immediately right after preparation. The lentiviral vector used to introduce a Green Fluorescent Protein (GFP) transgene for the isolation of GFP^+^-EVs was produced employing the calcium phosphate method into 293FT packaging cells.

### 2.5. iPSC Generation

To induce reprogramming, we exposed pericytes and fibroblasts, at early passages, to fresh lentiviral medium 3 times at 12 h intervals. Lentiviral medium was then replaced with fresh medium. After a further 5 days, 1 × 10^3^ transduced cells/cm^2^ were plated on a feeder layer constituted of inactivated mouse embryonic fibroblast (iMEF). Cells were then cultured in iPSC medium composed of knockout DMEM (Life Technologies, Carlsbad, CA, USA), supplemented with 20% knockout Serum Replacement (Life Technologies), 20 ng/mL of basic fibroblast growth factor (bFGF; Life Technologies), 1% N-2 (Life Technologies), 2% B27 (Life Technologies), 2 mM Glutamax (Life Technologies), 100 µM Eagle′s minimum essential medium non-essential amino acid solution (MEM-NEAA, Life Technologies), 100 µM β-mercaptoethanol, 100 U/mL penicillin and 100 µg/mL streptomycin. After iPSC line expansion and characterization was carried out, cells were adapted to feeder-free condition, by seeding them on Geltrex matrix (Thermo Fisher Scientific) in Essential 8 medium (Life Technologies).

### 2.6. iPSC Multilineage Differentiation 

Cardiomyocyte differentiation was performed using STEMdiff Cardiomyocyte Differentiation Kit (StemCell Technologies, Vancouver, BC, Canada) according to the manufacturer’s instructions. Briefly, uniform undifferentiated iPSC colonies were harvested and seeded as single cells at 3.5 × 10^5^ cells per well in a 12-well format. After 48 h, the iPSC medium was replaced with Medium A to induce the cells toward a cardiomyocyte fate. On day 2, a full medium change was performed with fresh Medium B. On days 4 and 6, medium B was replaced with fresh Medium C. On day 8, medium was switched to cardiomyocyte Maintenance Medium with full medium changes on days 10, 12 and 14, to promote further differentiation into cardiomyocyte cells.

Neural differentiation was promoted plating 1 × 10^6^ cells/mL in Neural Induction Medium (Thermo Fisher Scientific) for 7 days. On day 8, iPSC-derived neural stem cells were harvested and expanded in Neural Expansion Medium (Thermo Fisher Scientific).

For endothelial differentiation, human iPSC cells were cultured in Roswell Park Memorial Institute medium (RPMI; Sigma-Aldrich, St. Louis, MO, USA) plus B27 medium with 6 μM CHIR99021 (CHIR; Sigma-Aldrich). On day 2, we replaced the medium with fresh RPMI supplemented with B27 and 2 μM CHIR. After 48 h, the medium was changed with EGM-2 medium supplied with vascular endothelial growth factor (VEGF; PeproTech, London, UK), bFGF, and SB431542 (Merck, Darmstadt, Germany). Every other day, the medium was changed with fresh EGM-2 medium supplied with VEGF, bFGF, and SB431542.

### 2.7. Isolation of MT-derived EVs

EVs were isolated from conditioned medium of C2C12 myoblasts, differentiated into myotubes, using HS, previously centrifuged at 100,000× *g* for 16 h at 4 °C for EV depletion. After 48 h of incubation in fresh medium, EVs were harvested and purified by differential centrifugation—cell debris and organelles were eliminated at 500× *g* for 20 min followed by another centrifugation at 3500× *g* for 15 min at 4 °C. EVs were pelleted by ultracentrifugation at 100,000× *g* for 70 min at 4 °C by L-80-XP ultracentrifuge (Beckman-Coulter, Brea, CA, USA). Finally, the pellet was washed with cold PBS (Phosphate Buffered Saline) in order to minimize sticking and trapping of non-vesicular materials. Purified EVs were used immediately after isolation.

### 2.8. Myogenic Differentiation by MT-Derived EVs

Human iPSCs with no differentiated colonies, expressing pluripotency markers were used for the differentiation process. The iPSCs were cultured under feeder-free conditions using Essential 8 medium on Geltrex matrix. A critical variable for the generation of robust myotube culture was the relative confluence at the onset of differentiation that it should be approximately 30%. After they were seeded for about 48 h, iPSCs were induced toward mesodermal commitment in Essential 6 medium (Life Technologies) and 1% ITS (insulin-transferrin-selenium) supplemented with 10 uM GSK3 inhibitor CHIR (Sigma-Aldrich). After 2 days, we withdrew CHIR from the culture medium. The mesodermal induction medium was replaced with fresh expansion medium composed of Essential 6 medium enriched with 1% ITS, 5 mM LiCl, 10 ng bFGF, 10 ng insulin-like growth factor 1 (IGF-1; Thermo Fisher Scientific) and 50 ug/mL MT-derived EVs. After further 4 days, LiCl was removed from the medium. During this period, cells underwent enhanced proliferation. Between days 8–10, cells reached confluence and were expanded using TryplE (Thermo Fisher Scientific) and Collagen Type I matrix coating (BD Biosciences). The final differentiation and maturation phase into myotubes took additional 2 weeks: by day 20, muscular progenitors were seeded on Collagen type I dishes; after cells reached confluence, growth factors and MT-derived EVs were removed from the medium, and cells were cultured only in Essential 6 medium supplemented with 1% ITS.

### 2.9. Flow Cytometry and Cell Sorting

Fluorescence-activated cell sorting (FACS) analysis on physical parameters (forward and side light scatter, FSC and SSC, respectively), was first performed in order to exclude small debris, while the LIVE/DEAD Fixable Dead Cell Stain (Invitrogen, Carlsbad, CA, USA) allowed for the discrimination between live and dead cells. Muscle pericytes were labelled with the following conjugated antibodies: anti-alkaline phosphatase-Cy5 (BD Pharmingen), anti-CD45-FITC/CD14-PE (BD Biosciences, San Jose, CA, USA), anti-NG2-PE (BD Pharmingen), anti-CD56-APC (NCAM; BD Biosciences), anti-CD146-Cy5 (MCAM; R&D Systems, Minneapolis, MN, USA), anti-PDGF-R-beta-FITC (R&D Systems), and anti-CD44-APC (BD Pharmingen). Skin fibroblasts were characterized by staining with anti-CD90-FITC (BD Pharmingen). iPSC-derived skeletal muscle progenitor cells were stained with primary antibodies: PAX3 (Thermo Fisher Scientific), MyoD1 (Abcam, Cambridge, UK), PAX7 (DHSB), MyoG (Clone F5D, eBioscience, San Diego, CA, USA), and myosin heavy chain (Clone MF20; R&D Systems) (Abcam), followed by staining with the FITC-conjugated secondary antibody (R&D System). All antibodies were diluted in accordance with the manufacturers’ instructions. Fluorescence intensity for surface antigens and intracellular cytokines was detected by flow cytometry using a BD FACS Canto II analyzer. Flow data were analyzed with the FACSDiva 6.1.2 software (Becton Dickinson, Franklin Lakes, NJ, USA) and the FlowLogic software (Miltenyi Biotec, Bergisch Gladbach, Germany).

The ALP^+^/CD56^−^ subpopulation was sorted by FACSAria II Cell Sorter (Becton Dickinson) and subsequently characterized by FACS analysis for the expression of pericyte markers (as listed above) following 2 passages in vitro.

To detect and analyze surface EVs markers by FACS analysis, we bound them to 4 μm aldehyde sulphate latex beads (Thermo Fisher Scientific) overnight at 4 °C in rotation. EV-coated beads were then incubated with fluorochrome-conjugated antibodies CD63-APC (eBioscience) and CD81-PE (Invitrogen), and diluted in accordance with the manufacturers’ instructions. A “beads only” control sample was used to set gating parameters.

For EV internalization, we labelled the purified vesicles isolated from C2C12 with 5 μg/mL CellMask Deep Red plasma membrane stain (Molecular Probes, Eugene, OR, USA) and 5 mM CellTrace Violet (Invitrogen) at 37 °C for 30 min. The labeled EVs were washed in PBS and ultra-centrifuged at 100,000× *g* at 4 °C for 90 min, suspended in differentiating medium and used to treat the cells. After 48 h, we detected fluorescence on differentiating cells by flow cytometry.

### 2.10. Gene Expression Analysis

Total RNA was extracted using small RNA miRNeasy Mini Kit (Qiagen, Hilden, Germany). A total of 1 µg of total RNA was reverse-transcribed to cDNA using SuperScript VILO cDNA synthesis kit (Life Technologies). qRT-PCR were performed using SYBR Green Master Mix (Applied Biosystems, Foster City, CA, USA). Each sample was analyzed in triplicate using QuantStudio 6 Flex Real-Time PCR System Software (Applied Biosystems). The relative gene expression for pluripotency markers was expressed relative to a certified Episomal iPSC lineage (EpiPSC, Thermo Fisher Scientific), and normalized to Glyceraldehyde-3-Phosphate Dehydrogenase (GAPDH) (Table S1).

The expression of the lentiviral vector was assessed by qualitative RT-PCR according to standard procedure. Amplified products were separated by electrophoresis on a 1% agarose gel. Primers were designed to identify cMyc (one of the four human transcription factors included in the polycistronic lentiviral backbone—forward oligonucleotide) and WPRE (woodchuck hepatitis virus post-transcriptional regulatory element, a lentiviral-specific transgene—reverse oligonucleotide) (Table S1).

### 2.11. Immunohistochemistry and Immunocytochemistry

Cells were fixed in 4% paraformaldehyde for 20 min at room temperature, washed twice in PBS and blocked with 10% donkey serum (Sigma-Aldrich) for 30 min at room temperature. For intracellular immunostaining, we permeabilized cells for 10 min in 0.1% Triton X-100 (Sigma-Aldrich). Immunofluorescence assays were carried out with the following primary antibodies as follows: pericytes were stained with anti-PDGFRβ (1:100 Abcam), anti-α-smooth muscle actin (1:400, SMA; Dako, Santa Clara, CA, USA) and anti-NG2 (1:200, Millipore, Burlington, MA, USA); fibroblasts were labelled with anti-vimentin (1:50, Sigma-Aldrich); HUVEC were incubated with anti-von Willebrand factor (1:100, Abcam); iPSC pluripotency was verified by anti-OCT4, anti-SSEA4, anti-SOX2 and anti-TRA-1-60 (1:100, all from Invitrogen); iPSC-derived MT were identified by anti-myosin heavy chain (MHC; 1:100; R&D).Incubations with the secondary antibodies (1:100, Jackson ImmunoResearch Laboratories, West Grove, PA, USA) were performed for 1 hour at 37 °C. Cells were then counterstained using Vectashield Mounting Medium with DAPI (4′,6-diamidino-2-phenylindole). Fluorescence was detected by microscope (Axio Observer A1, Zeiss, Oberkochen, Germany).

Differentiation evaluation was assessed by immunofluorescence for MHC and fusion index scoring, defined as the ratio between the number of myosin heavy chain expressing myotubes with greater than 2 nuclei with respect to the total number of nuclei.

### 2.12. Western Blotting Analysis

EVs were also characterized by Western blotting for the expression of specific markers. EVs were lysed in radioimmunoprecipitation assay buffer (RIPA buffer), and supplemented with protease and phosphatase inhibitor cocktails (Roche Diagnostics GmbH, Mannheim, Germany). Proteins (30 μg) were separated on the Nupage Novex on 4–12% Bis-Tris Gel (Life Technologies) and transferred to a Polyvinylidene fluoride (PVDF) membrane Amersham Hybond (GE Healthcare Biosciences, Piscataway, NJ, USA). Membranes were incubated overnight at 4 °C with primary antibodies anti-CD81, anti-CD63, anti-αHSP70 (ExoAb Antibody Kit, System Biosciences, Palo Alto, CA, USA), anti-TSG-101 (Thermo Fisher Scientific), anti-MyoD1 (Abcam), anti-MHC (R&D) followed by peroxidase-linked anti-rabbit IgG or anti-mouse IgG secondary antibodies (GE Healthcare Life science) for 1 h at room temperature. Specific protein bands were detected with Pierce ECL Western Blotting Substrate (Thermo Fisher Scientific).

### 2.13. In Vivo Studies

Two-month-old male αSGKO/SCIDbg mice (n = 5) were anesthetized with an intramuscular injection of physiologic saline (10 mL/kg) containing ketamine (5 mg/mL) and xylazine (1 mg/mL) and then 5 × 10^5^ PC-derived iPSCs were injected into the Tibialis Anterior muscle (TA), according to standardized procedures [5]. Mice were sacrificed 20 days after implantation for morphological analysis. Experiments on animals were conducted according to the rules of good animal experimentation I.A.C.U.C. no 432 of 12 March 2006 and under Italian Health Ministry approval no. 228/2015-PR. In vivo experiments were conducted in accordance with the principles of the 3Rs (replacement, reduction and refinement).

### 2.14. Engrafted Human Muscular Cell Identification

Human differentiated iPSCs were identified by immunofluorescence for anti-human lamin A/C (1:100, SIGMA). Anti-laminin (1:100, SIGMA) was used to identify the fibers. The images were obtained by confocal laser scanning microscope.

### 2.15. Statistical Analysis

Statistical significance of the differences between means was assessed by one-way analysis of variance (ANOVA), followed by the Student-Newman-Keuls test, to determine which groups were significantly different from the others. When only two groups had to be compared, we used the unpaired Student’s t-test. *p* < 0.05 was considered significant. Values are expressed as means ± standard deviation (SD). All the analyses were performed using Graph-Pad PRISM 7 and 8.

## 3. Results

### 3.1. Pericyte and Fibroblast Isolation and Characterization

**Figure 1:**
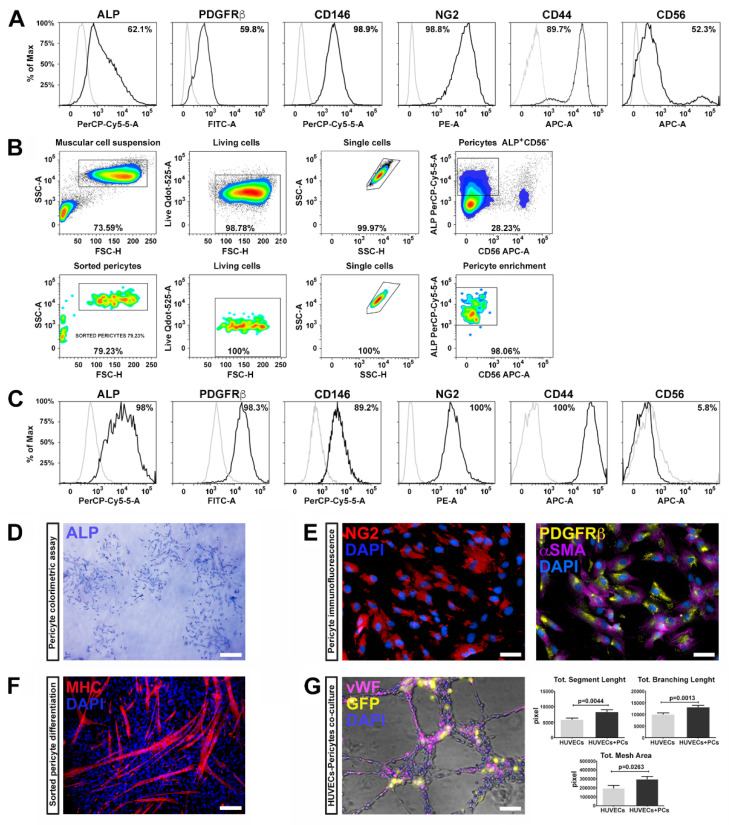
Pericyte characterization. (**A**) Representative histograms indicating the percentage of alkaline phosphatase (ALP^+^), platelet derived growth factor receptor-beta (PDGFRβ^+^), MCAM, melanoma cell adhesion molecule (CD146^+^), (αSMA^+^), Neuron/glial antigen 2 (NG2^+^), HCAM, homing cell adhesion molecule (CD44^+^) and NCAM, neural-cell adhesion molecule (CD56^+^) positivity (black peaks) determined by flow cytometry in pre-sorted cells isolated from muscular biopsy (n = 4). Matched isotypes were used as negative controls (grey peaks). (**B**) Representative gating strategy for ALP^+^ and CD56^−^ cell sorting (n = 3). Cells were first gated for cell size (side light scatter SSC-A vs. forward light scatter FSC-H) and vitality (Live Qdot-525-A). The muscular cell gate was further analyzed for singlets (SSC-A vs. SSC-H) and their expression for ALP and CD56. Pericytes, ALP^+^ and CD56^−^ were then sorted from this gated population. The lower set of four plots confirmed the efficiency of the sorting. (**C**) Representative post-sorting histograms for key pericyte markers after two passages in vitro, indicating an enhanced expression of ALP, NG2, PDGFRβ, CD146, and CD44. (**D**) Sorted pericytes stained for ALP showing fibroblast colony-forming units (CFU-F) when seeded at low confluence. Scale bar represents 300 μm. (**E**) Immunofluorescence labeling for NG2 (red) and the co-staining for PDGFRβ (green) and αSMA (magenta) on sorted ALP^+^CD56^−^ cells. Nuclei were stained with DAPI. Scale bar represents 50 μm. (**F**) Representative fluorescence image for myosin heavy chain (MHC) (red), validating the differentiation of sorted pericytes toward skeletal muscle phenotype. Scale bar represents 100 μm. (**G**) Illustrative images of human umbilical vein endothelial cells (HUVEC) in co-culture with pericytes displaying the formation of capillary-like networks with HUVEC labeled for von Willebrand factor (vWF; magenta), and GFP^+^ pericytes. Nuclei were identified by DAPI (blue). Scale bar represents 100 μm. Tubular structures were photographed at 5× magnification and quantified by the angiogenesis analyzer ImageJ tool. Total segment length, total mesh area and total branching length exhibited significant differences between HUVEC alone and in co-culture with pericytes, as shown in the graphs.

Pericytes were isolated from three healthy human skeletal muscle biopsies and characterized by flow cytometry using a specific panel of markers according to previous studies [6]. The harvested cells highly expressed well-known pericyte markers, such as ALP (alkaline phosphatase), PDGFRβ (platelet derived growth factor receptor-beta), CD146 (MCAM, melanoma cell adhesion molecule), NG2 (Neuron/glial antigen 2), and CD44 (HCAM, homing cell adhesion molecule) (Figure 1A) as well as CD56 (NCAM, neural-cell adhesion molecule), a glycoprotein specifically expressed in muscle by human satellite cells [6,34].

Skeletal muscle resident PCs, expressing ALP, represent a myogenic cell compartment, distinct from satellite cells, capable of promoting myofiber regenerating [6]. Therefore, we selected pericytes with myogenic potential by fluorescence-activated cell sorting (FACS), combining the cell surface markers ALP and CD56. We enriched the pericyte population selecting the fraction ALP^+^CD56^−^, which represented the 28% of the total population (Figure 1B).

After expansion and before reprogramming, ALP^+^CD56^−^ subpopulation was analyzed for the expression of the canonic muscular pericyte markers—almost the totality of the tested cells expressed ALP, PDGFRβ, CD146, NG2, and CD44, while the expression of CD56 was dramatically reduced (Figure 1C), suggesting that ALP^+^CD56^−^ fraction retains pericyte features.

These results are in line with previous studies in which pericyte identification was performed through the combination of NG2, PDGFβ, and CD146 markers [35,36]. PC phenotype was further confirmed by the ALP colorimetric assay (Figure 1D) and immunofluorescence positivity for NG2, PDGFRβ, and αSMA (Figure 1E).

We further examined the myogenic potential of ALP^+^CD56^−^ cells by measuring myosin heavy chain (MHC) expression, upon skeletal muscle differentiation, induced by cellular confluence and serum depletion [37]. After two weeks, ALP^+^CD56^−^ cells spontaneously differentiated into myosin positive multinucleated myotubes, as confirmed by the expression of MHC (Figure 1F). These results indicate that pericytes possess myogenic potential, along with supporting vessel formation and angiogenesis.

PCs are crucial in several phases during angiogenesis and vascular homeostasis, regulating the germination of the capillaries and the stabilization of the vessels. We therefore evaluated the ability of ALP^+^CD56^−^ cells to generate networks and to cooperate with endothelial cells (HUVECs) to form capillary-like structures. For this purpose, we transduced cells with a lentivirus expressing GFP, co-cultured with HUVECs (GFP^+^ ALP^+^CD56^−^ cells/HUVECs in a 1:4 ratio), and assembled on Matrigel for 6 h. We found that the ALP^+^CD56^−^ cells significantly enhanced capillary-like structure formation of HUVECs. Indeed, PCs co-cultivated with HUVECs displayed higher segment total length, total mesh area and total branch length compared to HUVECs cultured alone (Figure 1G). These results demonstrate that pericytes isolated from skeletal muscle maintain their ability to support vessel formation and myogenic potential after isolation, sorting and expansion procedures.

Epigenetic memory inherited from their original tissue have been demonstrated to influence the iPSC differentiation potential [29], suggesting that pericyte myogenic and angiogenic potential could be advantageous in tissue regeneration. Hence, we isolated human adult skin fibroblasts from the same donor of pericytes in order to compare the capability of iPSCs derived from pericytes (PC-derived iPSCs, ALP^+^CD56^−^ subpopulation) and fibroblasts (FB-derived iPSCs) to re-differentiate into muscle cells. Skin fibroblasts, isolated by enzymatic digestion, were characterized by immunofluorescence (Figure S1) and FACS analysis (Figure S1) for the expression of vimentin and CD90.

### 3.2. iPSC Generation and Characterization

**Figure 2:**
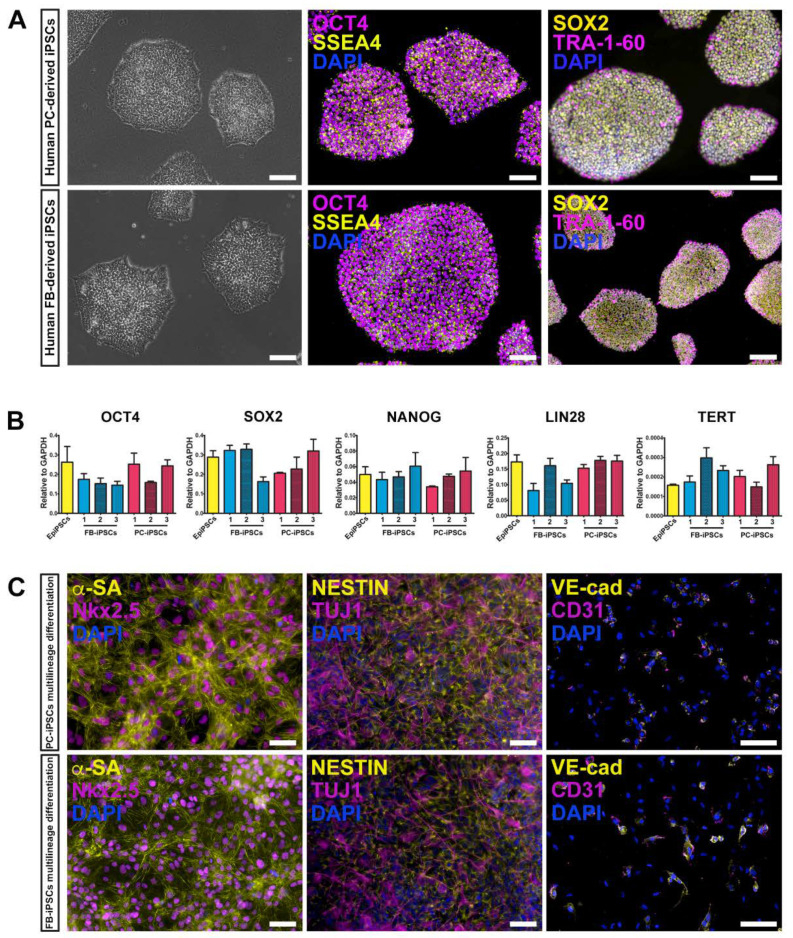
Characterization of human pericyte (PC)- and fibroblast (FB)-derived iPSCs. (**A**) Morphology of PC-derived iPSCs (left upper panel) and FB-derived iPSCs (left lower panel) cultured in feeder-free conditions. Scale bar represents 100 μm. Immunofluorescence labeling for the expression of the pluripotent markers SSEA4 (yellow), OCT4 (magenta; middle panels), SOX2 (yellow), and TRA-1-60 (magenta; right panels) on PC- and FB-derived iPSCs (PC-iPSCs and FB-iPSCs, upper and lower panels respectively). Nuclei were stained with DAPI. Scale bar represents 100 μm and, for the lower right image, 200 μm. (**B**) Quantitative RT-PCR analysis for the expression of the embryonic genes, OCT4, SOX2, NANOG, LIN28, and TERT—in PC- and FB-derived iPSC lines, derived from three donors (named FB-/PC-derived iPSCs 1, 2, 3), compared to the certified Episomal iPSC lineage (EpiPSC), used as control. Results were normalized to GAPDH. (**C**) Representative fluorescence images demonstrating multilineage differentiation capacity of PC- and FB-derived iPSCs toward cardiomyocyte (alpha-sarcomeric actin–α-SA, Nkx2.5), neuronal (Nestin, TUJ1) and endothelial (CD31, VE-cadherin–VE-cad) lineages. Nuclei were stained with DAPI (blue). Scale bars represent 50 μm (left and middle panels) and 100 μm (right panels).

We generated human muscular PC-derived iPSCs and skin FB-derived iPSCs from the same donor (n = 3 donors) using a polycistronic vector harboring the four Yamanaka factors (OCT4, Sox2, Klf4, cMyc) [33,38]. Colonies were initially expanded on a feeder layer of inactivated mouse embryonic fibroblasts (iMEF) and then adapted to feeder free conditions replacing iMEF with geltrex matrix (Figure 2A, left panels). iPSC colonies expressed typical pluripotent markers, including OCT4, SSEA4, SOX2, and TRA-1-60, as assessed by immunofluorescence staining (Figure 2A, middle and right panels). These results were confirmed by quantitative real-time PCR (qRT-PCR) for the expression of OCT4, SOX2, NANOG, LIN28, and TERT genes. Certified Episomal iPSC lineage (EpiPSC, Thermo Fisher Scientific) was used as control (Figure 2B).

Qualitative RT-PCR was conducted to verify the silencing of the exogenous transgenes in PC- and FB-derived iPSCs (Figure S2).

In addition, both derived iPSC lines retained the ability to differentiate toward multiple lineages, including cardiomyocytes, neuronal precursors, and endothelial cells (Figure 2C).

### 3.3. MT-derived EV Characterization

EVs represent an important vector of intercellular communication, acting as vehicles for the transfer of cytosolic factors, proteins, lipids and RNA [39]. EV cargo is cell-type specific, and the molecular composition reflects specific functions of the donor cells. 

EV secretion and extracellular signaling occurs during muscle differentiation, repair and regeneration [25,40]. Both myoblasts and myotubes release EVs, but their contribution in muscle physiology and specific biological functions on recipient cells have not been fully elucidated.

Proteomic analysis of muscle-derived EVs revealed that, in addition to proteins involved in their biogenesis, EVs also contain functionally relevant proteins such as myogenic growth factors and contractile proteins [26,40,41].

On the basis of these premises, we have exploited the capacity of EVs, secreted during myotube formation of skeletal myoblasts (C2C12), to promote the differentiation of iPSCs into the myogenic lineage.

**Figure 3:**
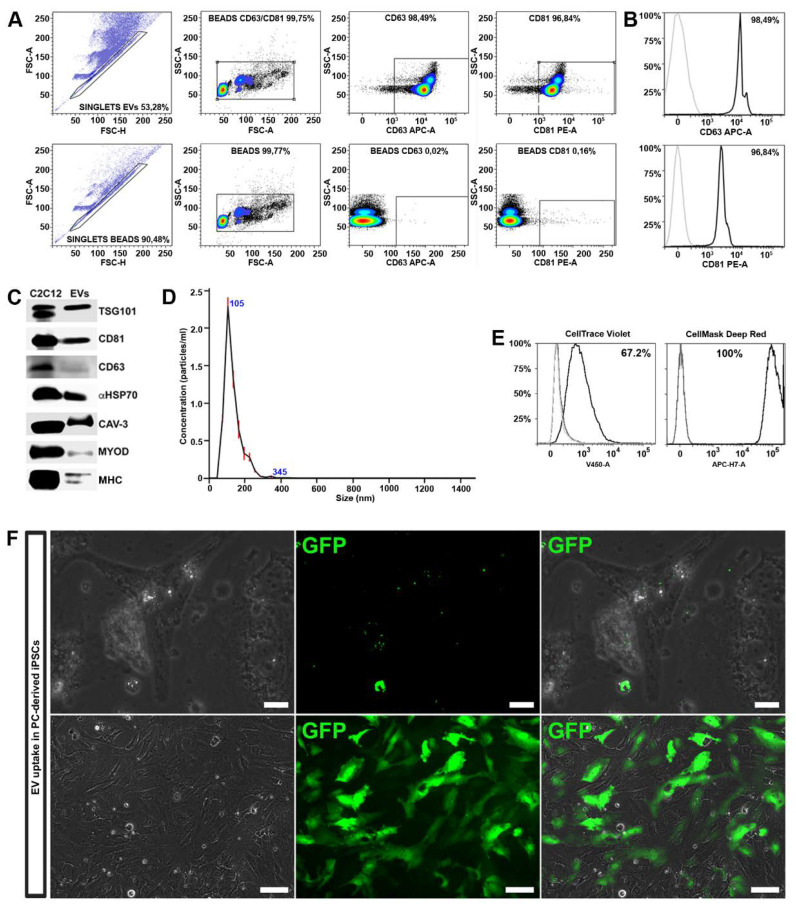
Extracellular vesicle (EV) characterization and uptake. (**A**) Sample gating strategy indicating the percentage of CD63^+^ and CD81^+^ in myotube (MT)-derived EVs coated with beads (n = 3). In the upper plots, singlets and subsequently EVs were selected according to physical parameters (FSC-A vs. FSC-H and SSC-A vs. FSC-A, respectively) are shown. Fluorescent intensity signal for CD63 and CD81 was detected on gated EVs (SSC-A vs. APC-A and SSC-A vs. PE-A, respectively). Beads alone were used as control (lower four scatter plots). (**B**) Representative histograms displaying the percentage of EV specific markers, such as CD81 and CD63, determined by flow cytometry in purified MT-derived EVs. Matched isotypes were used as negative controls (grey peaks) (**C**) Western blot for specific the expression of EV markers, such as TSG101, CD81, CD63, aHSP70, and skeletal muscle markers, such as CAV-3, MYOD, MHC, in C2C12-derived myotubes and C2C12 myotube-derived EV lysates. (**D**) Size distribution profile of MT-derived EVs (n = 5). (**E**) Representative histograms, determined by flow cytometry, displaying the percentage of positive cells (black) after the treatment with EVs stained for cell trace violet or cell mask deep red. Non-treated cells (light grey peaks) and cells treated with unstained EVs (dark grey peaks) were used as controls. (**F**) Green spots indicate the presence of fluorescent (GFP^+^) EVs, derived from GFP transduced myotubes, in the cytoplasm of the recipient differentiating iPSC after 48 h of exposure (upper panels; scale bars represent 20 μm); GFP^+^ cells 10 days after GFP^+^ MT-derived EV exposure (lower panels; scale bars represent 100 μm), demonstrating that GFP was transferred through the EVs into the recipient cells.

EVs were purified from conditioned media of C2C12-derived MTs cultured in 2% EV-depleted horse serum. EV isolation was performed by differential centrifugations according to well-established protocols [42]. EVs were further analyzed by FACS analysis for the expression of CD81 and CD63. We found that MT-derived EVs were enriched in membrane-bound tetraspanins CD63 and CD81, which are common markers for EV subsets released from most cell types (Figure 3A,B). The expression of CD81 is observed on vesicles of various sizes indicating that multivesicular endosomes in muscle cells contain intraluminal vesicles of heterogeneous sizes [27,41]. The expression of proteins associated with EVs, such as tumor susceptibility gene 101 (TSG101), α heat shock 70 kDa protein 4 (HSP70), CD81 and CD63 was further confirmed by Western blot analysis.

We found that MT-derived EVs exhibited specific membrane proteins associated with mature muscle tissue, such as caveolin 3 (Cav3), expressed only during the late stage of differentiation, and MHC, a differentiated myotubes marker (Figure 3C). Interestingly, MyoD, a transcription factor implicated in myogenesis, was also detected within the EV cargo.

Finally, the purity, size and concentration of the MT-derived EVs were determined by nanoparticle tracking analysis (NTA), using instrument-optimized analysis settings in NTA 3.1 build 54 software. The results showed a mean size distribution, consistent with what is expected from a sample enriched in microvesicles and exosomes (130.3 ± 0.3 nm), and free from contamination by apoptotic bodies (> 1 μm) (Figure 3D).

### 3.4. Detection of the MT-derived EV Uptake

To exert their functional influence, EV cargo must be internalized within the cell in adequate concentrations. As first step, in order to verify if EVs can transfer their contents to differentiating iPSCs, we performed EV uptake assays. For this experiment, we employed two different methods: FACS analysis and immunofluorescence microscopy. Upon isolation, MT-derived EVs were marked either with cell mask deep red or with cell trace violet. As controls we used untreated cells and cells exposed to unstained EVs. After 48 h, FACS analysis revealed an increased fluorescence of both tracers in recipient cells, indicating a successful uptake (Figure 3E).

In order to demonstrate the EV cargo delivery, we transduced C2C12 cells with a lentivirus expressing the GFP protein, and subsequently differentiated into myotubes. The EVs released by GFP^+^ myotubes were collected and used to treat the differentiating iPSCs. After 48 h, a fluorescent signal was detected in the cytoplasm of cells undergoing differentiation (Figure 3F, upper panels).

Cells were treated with EVs released by GFP^+^ myotubes every other day and, after 10 days, approximately 40% of cells expressed GFP in the cytoplasm (Figure 3F, lower panels) indicating a high functional cargo delivery to the recipient cells. EVs may enter into a cell via more than one route, depending on proteins and glycoproteins found on the surface of both the vesicle and the target cell [43]. The mechanisms responsible for MT-derived EVs delivery have not yet been determined highlighting the need for further research [44].

Considering these results, we have shown that EVs target iPSCs via delivery of effector molecules directly affecting their phenotype and functions.

### 3.5. Myogenic Differentiation by MT-derived EVs

MT-derived EVs express specific cell-adhesion molecules on their surfaces (ITGB1, CD9, CD81, CD44, Myoferlin) that are involved in the recognition and adhesion during the process of myoblast fusion [45,46,47].

Further, MT-derived EVs contain functionally active proteins of the G-protein family, which are involved in many cellular processes including myogenesis [48].

Wnt signaling and its modulation via GSK3 inhibitors, such as CHIR99021 (CHIR), is essential in the mesoderm induction and to obtain a reliable, reproducible and efficient myogenic differentiation protocol [18,19,49].

**Figure 4:**
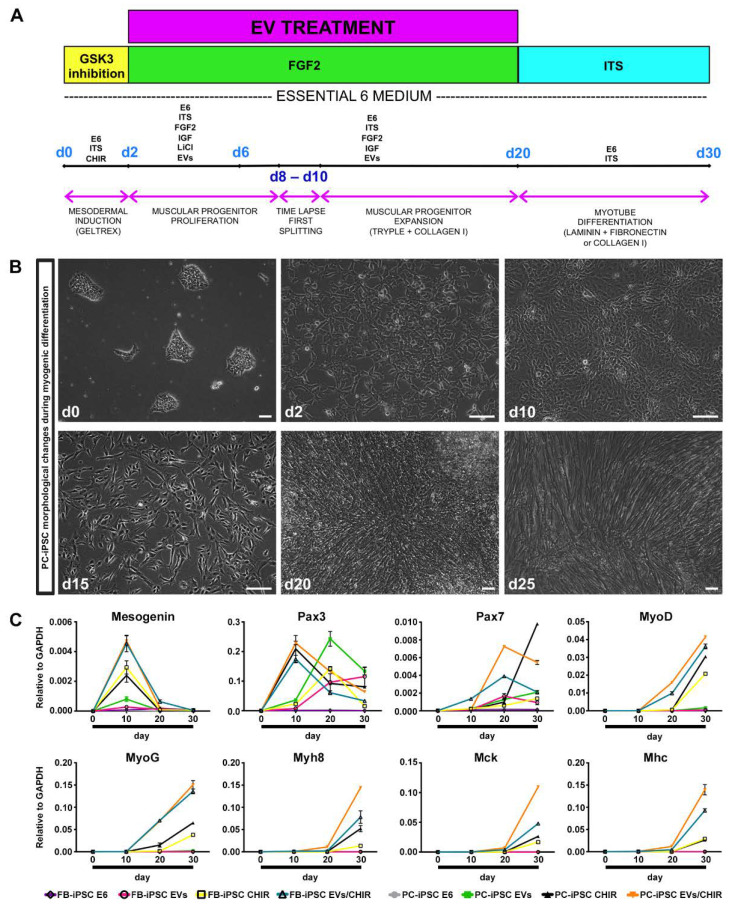
Myogenic differentiation by MT-derived EVs. (**A**) Schematic diagram of the myogenic differentiation procedure: 30% confluent iPSCs were differentiated to early mesoderm using CHIR for 48 h, subsequently proliferation and expansion of muscular progenitors were stimulated utilizing EVs, FGF2, IGF, and the myotube-like cell maturation was induced, removing growth factors from the culture medium. (**B**) Morphological changes of PC-derived iPSC colonies during skeletal muscle differentiation. Scale bars represent 100 μm. (**C**) Representative qRT-PCR for the expression of early (Mesogenin, Pax3, Pax7, MyoD, MyoG) and late (MyH8, MCK, MHC) skeletal muscle genes. The analysis was performed at different time points (d0, d10, d20, d30) in the following conditions: FB- and PC-derived iPSCs not treated with EVs or CHIR (FB-iPSCs E6 and PC-iPSCs E6), exposed to CHIR or EVs only (FB-iPSC EVs, FB-iPSC CHIR, PC-iPSC EVs, PC-iPSC CHIR) and treated with both, EVs and CHIR (FB-iPSC EVs/CHIR and PC-iPSC EVs/CHIR) (n = 3 donors). Results were normalized to GAPDH.

On the basis of these findings, we developed a method to induce a robust differentiation of iPSC toward skeletal muscle phenotype combining MT-derived EV cargo and the chemical modulator CHIR for paraxial mesoderm-like muscle progenitor commitment (Figure 4A).

PC- and FB-derived iPSCs, derived from three donors, were divided in four groups and treated as follows: (i) group 1, PC-/FB-derived iPSCs cultured in differentiation medium; (ii) group 2, PC-/FB-derived iPSCs cultured in differentiation medium augmented with EVs; (iii) group 3, PC-/FB-derived iPSCs cultured in differentiation medium enriched with CHIR; and (iv) group 4, PC-/FB-derived iPSCs cultured in differentiation medium supplemented with EVs and CHIR.

Cells were treated with 10 uM CHIR for 48 h to induce the expression of paraxial mesoderm genes. Consistent with other studies [18,19], iPSCs from both sources presented evident morphological changes losing the typical ES-like morphology after 24 h of treatment (Figure 4B). Following replacement of CHIR with FGF2, cells underwent proliferation and reached full confluence approximately at day 8–10.

Following 48 h of treatment, CHIR was removed and, in order to enhance the myogenic differentiation, the media were enriched with freshly MT-derived EVs (50 μg/mL) and replenished every 2 days with fresh vesicles until the formation of myoblast-like cells. Between days 15 and 25 paraxial mesoderm-like muscle progenitors can be either expanded or terminally differentiated upon withdrawal of all the growth factors from the medium. The outcome of myotube formation was greater when myoblast-like cells were passaged 2–3 times and consequently exposed longer to MT-derived EVs.

We have analyzed the expression of genes related to mesoderm induction and myogenic differentiation at different time points through qRT-PCR experiments (Figure 4C).

In PC- and FB-derived iPSCs treated with the CHIR/EV combination, pre-myogenic mesoderm genes, such as Mesogenin, Pax3, and Pax7, were upregulated between day 10 and day 20. The myogenic regulatory factor MyoD and MyoG started to be expressed around day 10 and increased up to day 30. Finally, the mature myocyte genes, Myh8 (myosin heavy chain 8), MCK (muscle creatine kinase) and MHC, were detected after day 20 and augmented until the end of the differentiation confirming the increase in differentiation and maturation of the iPSCs treated with both factors.

Cells cultured in differentiation medium were negative for the expression of myogenic genes, while iPSCs treated with EVs exhibited only pre-myogenic genes (Pax3 and Pax7), indicating that the MT-derived EVs are not sufficient, at least within 30 days, to induce myotube differentiation. Lastly, cells exposed to CHIR presented a myogenic inclination similar, but less efficient, to PC-/FB-derived iPSCs treated with the CHIR–EVs mishmash.

**Figure 5:**
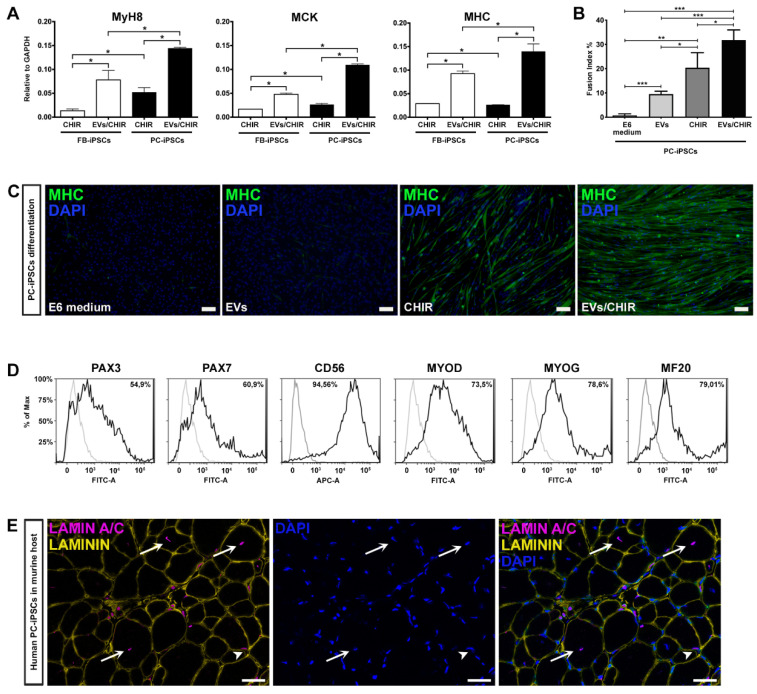
iPSC-derived MT characterization. (**A**) Representative qRT-PCR for the expression of Myh8, MCK and MHC after 30 days of differentiation. The combination CHIR-EVs induced a greater differentiation compared to CHIR treatment and PC-derived iPSCs possessed a greater propensity to differentiate into myotubes compared to FB-derived iPSCs. Results were normalized to GAPDH. (**B**) Fusion index was calculated as the percentage of nuclei within myosin heavy chain–positive myotubes (≥2 nuclei) divided by the total number of nuclei. A minimum of five random fields at 10X magnification were counted. (**C**) Immunofluorescence labeling for MHC (green), validating the differentiation capacity of PC-derived iPSCs toward skeletal muscle phenotype after 30 days of differentiation. The images represent sequentially: PC-derived iPSCs without treatment, exposed to EVs alone, to CHIR only and to the EV–CHIR cocktail. CHIR in combination with MT-derived EVs induced a higher expression of the skeletal marker (n = 3 donors). Nuclei were stained with DAPI. Scale bar represents 100 μm. (**D**) Representative histograms indicating the percentage of PAX3, PAX7, CD56, MYOD, MYOG, and MF20 (black peaks) determined by flow cytometry in PC-iPSC-derived MTs exposed to CHIR and MT-derived EVs after 30 days of differentiation. Matched isotypes were used as negative controls (grey peaks) (n = 3 donors). (**E**) Representative immunofluorescence against human lamin A/C (magenta) and laminin (yellow) on mouse anterior tibialis (TA) injected with human PC-derived iPSC exposed to CHIR-EVs (n = 3 donors). Arrows indicate center-nucleated human derived myofibers, arrowhead labelling integrated human nuclei into mature host muscle fiber. Nuclei were stained with DAPI. Scale bars represent 25 μm.

Our method proved that the combination CHIR-EVs induces a higher differentiation compared to CHIR treatment (Figure 5A).

To verify whether PC-derived iPSCs, generated from the three donors, possessed a greater propensity to differentiate into myotubes compared to FB-derived iPSCs, we matched the expression of Myh8, MCK, and MHC by qRT-PCR analysis at day 30 of differentiation (Figure 5A). The expression of mature myogenic genes resulted as being higher in differentiated PC-derived iPSCs.

To further determine the role of the EVs/CHIR combination we calculated the fusion index, which was significantly greater compared to the index obtained with the separate exposure of EVs or CHIR (Figure 5B). Consistently, also the cell number expressing MHC was also significantly greater in those generated using the CHIR–EVs cocktail compared to the other treatments (Figure 5C).

The ability of PC-derived iPSCs to generate differentiated cells presenting a characteristic mark of myogenesis, was further confirmed by FACS analysis. Myogenic regulatory factors, Pax3, Pax7, CD56, MYOD, MYOG, and MF20, were used to characterize the expression of myogenic proteins (Figure 5D). At day 30 roughly 80% of the cells were positive for the myocyte mature marker MF20.

Finally, after 25 days of CHIR–EV differentiation, PC-iPSC-derived muscular cells, were implanted in vivo in a limb girdle muscular dystrophy murine model, namely, SCID-Beige α–Sarcoglycan null mice (αSGKO/SCIDbg) [5] in order to evaluate their engraftment capabilities. Three different PC-derived iPSC clones were intramuscularly injected (5 × 10^5^ cells/injection) into the anterior tibialis (TA) muscle of αSGKO/SCIDbg, showing a sufficient engraftment and integration into regenerating muscle fibers revealed by the labelling of human derived cells by immunofluorescence against human specific lamin A/C antibody (Figure 5E).

## 4. Discussion

In the last few years, there has been a growing interest in the use of iPSCs as a source of myogenic progenitors for cell-based treatment in muscle degenerative diseases. iPSCs overcome several of the limitations related to the use of adult myoblast therapy, such as, non-invasive biopsy for cell isolation, unlimited proliferative capacity in vitro, and a tool for the in vitro correction of genetically mutated somatic cells derived from patients affected by dystrophies [14,15,16,19,50,51,52]. Moreover, it is well known that iPSCs maintain the epigenetic memory of the original cell source, influencing the re-differentiation toward the same lineage [30,53]. Pericytes resident in adult skeletal muscle have shown remarkable angiogenic and myogenic differentiation capacities in vitro and in vivo [7]. Hence, in this study, exploiting the advantage of the “epigenetic retention”, we proposed the isolation of human derived muscular pericytes to verify whether PC-derived iPSCs are more prone to differentiate into muscular cells compared to FB-derived iPSCs.

To date, the strategies used for iPSC myogenic differentiation are subdivided into two approaches: transgenic (by forced expression of Pax7 or MyoD) or non-transgenic (co-culture, EBs, small molecules) [15,16,51]. The former consists in the differentiation via overexpression of myogenic transcription factors, obtaining efficient iPSC differentiation into myogenic lineage in a relatively short amount of time [15,16,51]. However, forced expression of skeletal master genes drastically reduces the proliferative capacity of skeletal muscle progenitor cells, hindering the molecular mechanisms leading to myogenic differentiation (important for disease modeling in vitro), as well as their in vitro and in vivo self-renewal. Another point to be considered is the potential of insertional mutagenesis events due to the random integration, since these genes are commonly delivered using lentiviral vectors. Integrated virus genomes are frequently associated with chromosomal damage, rearrangements and deletions [54], making these approaches not suitable in terms of clinical applications. On the other hand, the non-transgenic methods use different sequential culture conditions, including cell sorting. These protocols are successful at producing myogenic progenitors capable of engrafting in vivo but lack reproducibility, besides being inefficient and time-consuming [55,56,57].

First-generation of transgene-free protocols employing different sequential culture conditions were not satisfactory and usually required post-differentiation selection to isolate expandable skeletal muscle progenitor cells [57]. In addition, in some described methods, iPSCs failed to express mature myogenic markers or form myotubes in vitro, underlying the poor efficiency of spontaneous myogenic differentiation method [55,56].

Improvements in muscle differentiation protocols were recently performed by employing a small molecule, CHIR99021, a GSK-3b inhibitor that activates Wnt signaling cascade, which plays a crucial role during early somite induction. These approaches have been proven to be efficient at boosting iPSC towards a myogenic pathway, leading to an ameliorated myotube differentiation in vitro [19,58,59].

Quite recently, considerable attention has been dedicated to extracellular vesicles (EVs) as important mediators of cell-to-cell communication. EVs can influence the behavior of recipient cells and are involved in many processes, including immune signaling, angiogenesis, stress response, proliferation, and cell differentiation [60,61,62]. Furthermore, EVs, through their paracrine signaling can be used in tissue engineering to modulate cell recruitment, differentiation, and proliferation [63]. Skeletal muscle cells secrete a large number of myokines and EVs that influence the growth, function and development of muscle tissue [26,27,41]. Transcriptome and proteome studies reported that EVs from C2C12 or human myoblasts are enriched in miRNAs and proteins that are implicated in myogenesis [22,27,40,41,64]. EVs isolated from differentiated C2C12 cells express specific cell-adhesion molecules on their surfaces (ITGB1, annexins CD56, CD9, CD81, CD44, Myoferlin), and other myokines (HGF, IGFI/II, FGF2, PDGF, TGFβ, myostatin) involved in myoblast fusion and muscle regeneration. [40,41,45,47,65]. In addition, differentially expressed miRNAs also contained in EVs during C2C12 myotube differentiation, such as miR-1, miR-133a, miR-133b, and miR-206, have been linked with muscle differentiation [64,66]. Interestingly, the Wnt signaling and Sirt 1 that are involved in muscle gene expression and differentiation, were predicted as the most significant targeted pathway by skeletal muscle EVs–miRNAs [64]. In the present study, we found that MT-derived EVs harbored myogenic factors that are crucial in enhancing the iPSC myogenic commitment. Despite several scientific papers reporting transcriptome and proteome profiling of EVs from skeletal muscle, the mechanisms and the key factors playing a major role in promoting skeletal muscle differentiation remain unclear. Evidence suggests that instead of single factors, the diverse EVs myogenic factors such as miRNAs or proteins, synergistically trigger skeletal muscle differentiation in recipient cells [22,26,27,40,41,64]. Further studies are required to define the biological role/function of single myogenic factors in MT-derived EVs.

Here, we have developed an efficient method for iPSC skeletal muscle differentiation combining defined factors with myogenic elements carried by EVs. We were able to generate a consistent number of iPSC-derived positive MHC skeletal muscle cells, evidencing differences in epigenetic signatures between PC-derived iPSCs and their counterpart FB-derived iPSCs. Indeed, quantitative PCR analyses performed at different timepoints and at the end of the differentiation, showed that PC-derived iPSCs are more prone to differentiate in skeletal muscle cells when compared to FB-derived iPSCs. Importantly, the myoblasts obtained with this approach could be expanded, and cryopreserved at all steps during expansion, without losing fusion competence. The obtained MT-like cells were positive for MyoD, MYOG, and MHC, and were able to actively participate in myogenesis in vivo. Transplanted myogenic progenitors demonstrated their ability to successfully engraft and integrate into TA muscle of αSGKO/SCIDbg, identified by the detection of centrally located human lamin A/C positive nuclei, a characteristic perceived only in fusion-competent myoblasts.

In this paper, we explored for the first time EVs as “physiological liposomes” enriched with myogenic factors that were able to trigger skeletal myogenesis in iPSC, raising exciting possibilities for therapeutic use. The use of EVs represent novel tools to deliver signaling molecules for treating muscle-wasting diseases that are better tolerated by the immune system. Additional studies are now required to investigate the role of EVs’ skeletal muscle content that may allow the development of better therapeutic approaches to promote skeletal muscle growth, differentiation and regeneration.

Our method may better recapitulate human developmental myogenesis providing inroads for patient-specific drug testing, disease modeling and regenerative approaches.

## Supplementary Materials

The following are available online at https://www.mdpi.com/2073-4409/9/6/1527/s1, Figure S1: Fibroblast characterization, Figure S2: Silencing of the exogenous factors, Table S1: List of human primers used for qRT-PCT analysis.
