# Supplementary material for: Extracellular Vesicles from Skeletal Muscle Cells Efficiently Promote Myogenesis in Induced Pluripotent Stem Cells
Source: Cells. 2020 Jun 23;9(6):1527. doi: 10.3390/cells9061527 (PMC7349204; doi:10.3390/cells9061527)
Supplement: Supplementary file 1 [file cells-09-01527-s001.pdf]

TABLE S1: human primers for quantitative and qualitative PCR

Gene		*Sequences (5′ to 3′)*
**OCT4 endogenous**	FW	AGTTTGTGCCAGGGTTTTTG
	REV	ACTTCACCTTCCCTCCAACC
**STEMCCA exogenous**	cMYC FW	GGAACTCTTGTGCGTAAGTCGATAG
	WPRE REV	GGAGGCGGCCCAAAGGGAGATCCG
**NANOG**	FW	CCCAAAGGCAAACAACCCACTTCT
	REV	AGCTGGGTGGAAGAGAACACAGTT
**SOX2 endogenous**	FW	AGCTACAGCATGATGCAGGA
	REV	GGTCATGGAGTTGTACTGCA
**LIN28**	FW	AGTAAGCTGCACATGGAAGG
	REV	ATTGTGGCTCAATTCTGTGC
**TERT**	FW	GGAGCAAGTTGCAAAGCATTG
	REV	TCCCACGACGTAGTCCATGTT
**Mesogenin**	FW	GTCCAGCGGAGGCGCAAAGC
	REV	GGTGTGCAGGGCATCTGCCAA
**PAX3**	FW	AATTCGGGAAAGGTGAAGAGG
	REV	TTCAGAGTCAATATCAGAGCCTTC
**PAX7**	FW	AAGAAGGCCAAACACAGCATCGAC
	REV	AGGTCAGGTTCCGACTCCACAT
**MYOD**	FW	TGCTCCGACGGCATGATGGACTA
	REV	TTGTAGTAGGCGCCTTCGTAGCAGTT
**MYOG**	FW	AATGCAGCTCTCACAGCGCCTC
	REV	TCAGCCGTGAGCAGATGATCC
**MYH8**	FW	CTCCATCTCTGACAATGCCTATC
	REV	AGTATTGGATGACACGCTTGG
**MCK**	FW	TGGAGAAGCTCTCTGTGGAAGCTC
	REV	TCCGTCATGCTCTTCAGAGGGTAGTA
**MYHC**	FW	TTCATTGGGGTCTTGGACAT
	REV	AACGTCCACTCAATGCCTTC
**GAPDH**	FW	GTGAAGGTCGGAGTCAACG
	REV	GGTGGAATCATATTGGAACATG

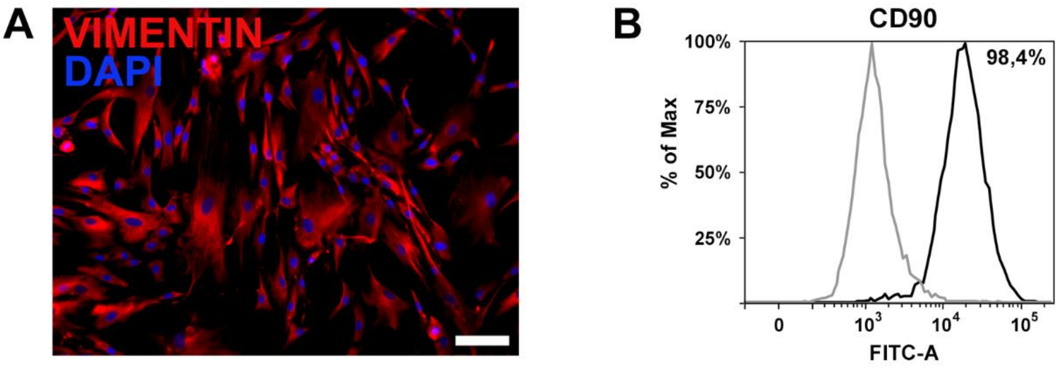

Figure S1. Fibroblast characterization. (**A**) Immunofluorescence labeling for vimentin (red) on skin fibroblast cells. Nuclei were stained with DAPI. Scale bar represents 100µm. (**B**) Representative histograms indicating the percentage of CD90$^+$ (black peak) determined by flow cytometry to identify fibroblast cell population (*n* = 4). Matched isotypes were used as negative controls (grey peak).

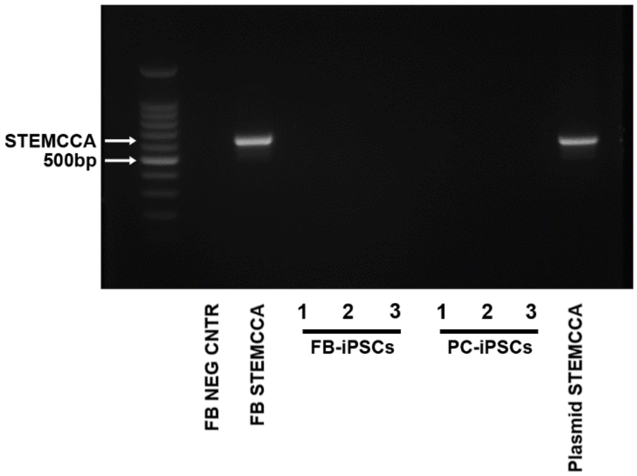

Figure S2. Silencing of the exogenous factors. Qualitative PCR reactions for the expression of the lentiviral vector; fibroblasts were used as negative control, while STEMCCA transduced fibroblasts and STEMCCA plasmid were used as positive controls.
